# Efficient Multi-Material Structured Thin Film Transfer to Elastomers for Stretchable Electronic Devices

**DOI:** 10.3390/mi13020334

**Published:** 2022-02-20

**Authors:** Xiuping Ding, Jose M. Moran-Mirabal

**Affiliations:** 1 Department of Chemistry & Chemical Biology, McMaster University, 1280 Main Street West, Hamilton, ON L8S 4M8, Canada; dingx12@mcmaster.ca; 2 Brockhouse Institute for Materials Research, McMaster University, 1280 Main Street West, Hamilton, ON L8S 4M8, Canada

**Keywords:** flexible electronics, wrinkling, shape-memory polymer, lift-off, hybrid structure, multilayer conductive films, wearable electronics

## Abstract

Stretchable electronic devices must conform to curved surfaces and display highly reproducible and predictable performance over a range of mechanical deformations. Mechanical resilience in stretchable devices arises from the inherent robustness and stretchability of each component, as well as from good adhesive contact between functional and structural components. In this work, we combine bench-top thin film structuring with solvent assisted lift-off transfer to produce flexible and stretchable multi-material thin film devices. Patterned wrinkled thin films made of gold (Au), silicon dioxide (SiO_2_), or indium tin oxide (ITO) were produced through thermal shrinking of pre-stressed polystyrene (PS) substrates. The wrinkled films were then transferred from the PS to poly(dimethylsiloxane) (PDMS) substrates through covalent bonding and solvent-assisted dissolution of the PS. Using this approach, different materials and hybrid structures could be lifted off simultaneously from the PS, simplifying the fabrication of multi-material stretchable thin film devices. As proof-of-concept, we used this structuring and transfer method to fabricate flexible and stretchable thin film heaters. Their characterization at a variety of applied voltages and under cyclic tensile strain showed highly reproducible heating performance. We anticipate this fabrication method can aid in the development of flexible and stretchable electronic devices.

## 1. Introduction

The implementation of stretchable electronics can enable new types of devices for a range of applications, like devices that can integrate with the human body for advanced therapeutic treatment, sensory skins for robotics, and wearable communication devices, which are impossible to achieve with conventional rigid electronics [1]. Flexible and stretchable components are key to the development of such devices for biomedicine, as has been shown in devices for intracardial and neural monitoring as well as human/machine interface integrated circuits [2]. Stretchable electronic devices should be mechanically robust to make it possible to conformally span curved surfaces and movable parts [3,4], and should be able to withstand large strains with no fracture or substantial degradation of their electrical properties [5]. For example, in a humanoid robot, areas covering the shoulders, elbows, and knees should be able to deform to 130–160% of their original dimensions [6]. Thin films have been used to implement stretchable electronic components because when the thickness of a thin film becomes 1/1000 of the desired radii of curvature, the tensile and compressive strains on the film during bending are small and the film becomes flexible and rollable [3,7].

Wrinkled structures, arising from the buckling mechanics of thin films, have been used to fabricate heterogeneous metal-elastomer stretchable electronic devices. The basis of this approach is that a supported thin film responds to an applied strain by buckling due to the stiffness mismatch between the rigid film and the compliant substrate. In such buckled materials, changes in amplitude and wavelength can occur reversibly when they are stretched or compressed [8]. For example, buckles with uniform 20–50 µm wavelengths have been created by thermally expanding a PDMS substrate, depositing a gold film by e-beam evaporation, and cooling down the system after gold deposition [9]. Similarly, stretchable and foldable silicon circuits have been fabricated by transferring the circuits from silicon-on-insulator wafer carrier substrates onto PDMS. In this approach, poly(methyl methacrylate) was spin coated as a sacrificial layer before the fabrication of the circuit arrays, and this layer was subsequently dissolved to release the ultrathin, flexible circuits during the transfer process [10]. 

The wrinkling of gold films by shrinking shape memory polymer substrates has been shown as an alternative cost-effective and simple method to make highly conductive stretchable electrodes. In this fabrication approach, thin gold films are deposited onto a shape-memory polymer substrate (e.g., polystyrene—PS) that is subsequently thermally shrunk [11], resulting in wrinkled thin films that can then be transferred from the rigid substrate to an elastomer like PDMS or Ecoflex [12,13]. In one example of this approach, the thin film transfer was done by dissolving a sacrificial photoresist layer under the gold film, manually lifting off the wrinkled film from the substrate, and depositing it onto partially cured PDMS. The advantage of a lift-off approach is that the photoresist layer can be quickly dissolved using acetone leaving behind minimal residues. A limitation of this method was the size of the transferred devices, as smaller, more fragile thin structures or thin films are difficult to transfer by hand without damage. A second method has been reported that avoids the use of a sacrificial layer and can faithfully transfer thin films patterned into complex shapes with small dimensions [12]. In this approach, the surface of a wrinkled gold film is treated with a solution of (3-mercaptopropyl)trimethoxysilane (MPTMS) to form a self-assembled monolayer that can covalently bond the film with a silicone elastomer mixture that is poured directly on top of it and cured. Through this method, highly stretchable wrinkled gold thin film electrodes were successfully fabricated. However, only a single layer gold film transfer was demonstrated, which limits the application of this method to more complex electronic devices and arrays, as many electronic devices are made in the form of multilayer composites. 

In this work, we present two solvent-assisted lift-off methods for the transfer of various wrinkled thin film materials and multilayer structures from rigid PS substrates to elastomeric substrates. The process begins with the fabrication of structured thin films by shrinking thin films deposited onto pre-stressed PS sheets. The wrinkled films are then treated by plasma oxidation or through the formation of a self-assembled monolayer, which promotes the covalent binding of the thin films to a PDMS elastomer that is cast on top and cured. Finally, either the sacrificial layer is dissolved, or the PS substrate is swelled and partially dissolved to remove the film/PDMS from the rigid PS substrate. These methods are simple and effective ways of fabricating composite structures for stretchable electronic devices. They can reliably transfer complex patterns of materials, and different architectures with varied dimensions. The ability to transfer complex multi-material composites can open the door to applications that require stretchable electrodes, dielectric, and semiconductor components. It can also solve interconnect problems, which are one of the key challenges for wearable electronic devices and arrays [14]. 

As proof-of-concept of this fabrication approach, we have implemented Au and ITO/Au/ITO stretchable resistive heaters and characterized their response to applied voltage and strain. Both types of heaters show fast heating response, high robustness, and remarkable resilience through stretching and relaxation cycles. This type of stretchable heater can address the need for portable, comfortable, functional materials for physiotherapy, where thermal therapy is frequently used to alleviate joint pain caused by obesity, aging, or workplace injuries [15]. Thin film wearable heaters with high mechanical robustness and good Joule heating performance can be used to overcome the mechanical rigidity and weight of current heat packs or wraps used for this method of therapeutic care. We anticipate that the fabrication of stretchable electronic devices through solvent-assisted lift-off and transfer of composite wrinkled thin films can be a simple and cost-effective way to producing highly stable and stretchable wearable electronics.

## 2. Materials and Methods 

### 2.1. Wrinkled Thin Film Fabrication

PS sheets (Graphix shrink film, Graphix, Maple Heights, OH, USA) were cut to desired sizes and cleaned by immersing them in isopropanol, ethanol, and 18.2 MΩ cm water (obtained from a Milli-Q Reference A+ Water Purification System, Millipore, Molsheim, France, subsequently referred to simply as “water”) bath sequentially and washing them on an orbital shaker (MAXQ 2000, Thermo Fisher Scientific, Waltham, MA, USA), for 5 min for each solvent. The PS sheets (shape memory polymer) used in this work are commercially available and biaxially pre-stressed. As a result, the side length of a PS sheet can be shrunken to 40% of its original size by simply heating the substrate above the glass transition temperature. Mask stencils were created by cutting self-adhesive vinyl (FDC-4300, FDC graphic films, South Bend, IN, USA) with a Robo Pro CE5000-40-CRP cutter (Graphtec America Inc., Irvine, CA, USA) to create the desired patterns for different devices. The self-adhesive vinyl stencil was then applied onto the clean PS substrates. For the samples lifted off with a sacrificial layer, positive photoresist (Microposit S1805, Shipley, Marlborough, MA, USA) was spin coated onto the masked PS substrate at 7000 RPM for 30 s and baked at 90 °C for 3 min to remove the solvent in the photoresist. The thickness of the spin-coated photoresist was measured with a surface profilometer (Tencor Alpha Step 200, KLA Corp., Milpitas, CA, USA). Thin films were deposited onto masked PS substrates by sputtering using a Torr Compact Research Coater CRC-600 manual planar magnetron sputtering system (New Windsor, NY, USA). A 99.999% purity gold target (LTS Chemical Inc., Chestnut Ridge, NY, USA), SiO_2_ target (Bayville Chemical Supply Company Inc., Deer Park, NY, USA), and ITO target (LTS Research Laboratories, Inc., Orangeburg, NY, USA) were used to deposit thin films of the respective materials. Gold films were sputtered using a DC (direct current) gun, and SiO_2_ and ITO films were deposited by RF (radio frequency) gun. After peeling off the vinyl mask, the PS substrate with deposited films were heated in an oven at 130 °C for 5 min to induce biaxial shrinking and flatted on a silicon wafer by annealing at 160 °C for 10 min. 

### 2.2. Lift-Off of Wrinkled Films from PS to PDMS

All samples with a gold film as the interface to be transferred to the elastomer were immersed in 5 mM (3-Mercaptopropyl) trimethoxysilane (MPTMS) (95% MPTMS, Sigma-Aldrich, MO, USA) aqueous solution for 1 h, rinsed with water and dried with nitrogen gas. Samples with SiO_2_/ITO films interfacing the elastomer were treated using a Harrick High Power Plasma Cleaner (PDC, Harrick Plasma Inc., Ithaca, NY, USA) for 1 min at high power (30 W). At the same time, the base of PDMS and curing agent (Sylgard 184 silicone elastomer kit, Dow Corning Corporation, Corning, NY, USA) were fully mixed in a 10:1 mass ratio and degassed in a desiccator with a mechanical vacuum pump until air bubbles were removed. The degassed PDMS was cast onto the surface-treated structured films, then put in an oven at 60 °C for 4 h to be cured. 

The cured PDMS was cut with a blade to expose the edges of PS substrate. Samples with and without photoresist were put into crystallization dishes with acetone and washed on an orbital shaker at 100 RPM. The photoresist was dissolved in acetone for approximately 1 h, at which point the PS substrate detached from the film/PDMS. For samples without a photoresist layer, the PS substrate was detached by swelling and softening PS in acetone for approximately 6 h, at which point the wrinkled thin films could be manually detached from the softened PS due to the strong chemical bonding between film and PDMS. However, a small amount of PS residue was visible on the surface of transferred films using this direct transfer method without photoresist. The PS residue was then removed by gently rinsing with toluene. The samples were then placed in the vacuum to extract excess solvent. Although toluene is effective at removing PS residues, the PDMS also swelled significantly in this solvent and PS residues were observed via SEM on samples with short toluene rinsing times. 

Hybrid films of Au/ITO/SiO_2_ were made by the fabrication procedure described and transferred with no sacrificial layer. 

### 2.3. Fabrication of Stretchable Thin Film Heaters 

The stretchable heater is comprised of conductive pads made of 50 nm-thick Au films, and heating elements made of 50 nm-thick Au films or 5 nm-thick ITO/50 nm-thick Au/5 nm-thick ITO composite films. Single deposition was conducted for the Au heater; for the ITO/Au/ITO heaters, both ITO layers were deposited on the heating element area, and Au was deposited on the whole area. The thin films were deposited onto pre-stressed PS substrates, and subsequently heated in an oven at 130 °C for 1 min to shrink the sample and produce wrinkled films. The transfer process used was the same as the procedure described in Section 2.2.

### 2.4. Characterization of Stretchable Thin Film Heaters 

To characterize the thermal and mechanical properties of the two types of thin film heaters, voltage was supplied by a source meter (2450 Source Meter, Keithley, Tektronix Inc., Solon, OH, USA). Thermal compound (ARCTIC MX-4, ARCTIC Ltd., Brunswick, Germany) was placed onto the heating element to absorb the heat and a thermocouple (T1 thermocouple, MS-6514 Thermometer, MASTECH-Group, Dongguan, China) was used to measure the temperature of the thermal compound. A home-built stretcher with the ability to control the stretch step and stretch distance was used to apply the external strain. The wires of the thermocouple were fixed onto a lab jack to ensure that the thermocouple would be reliably inserted into the thermal paste. The morphology of the thin films was characterized by scanning electron microscopy (SEM) imaging (JSM-7000F, JEOL Ltd., Tokyo, Japan), with a 10.0 kV acceleration voltage.

## 3. Results and Discussion

### 3.1. Thin Film Transfer with and without a Sacrificial Layer 

**Figure 1:**
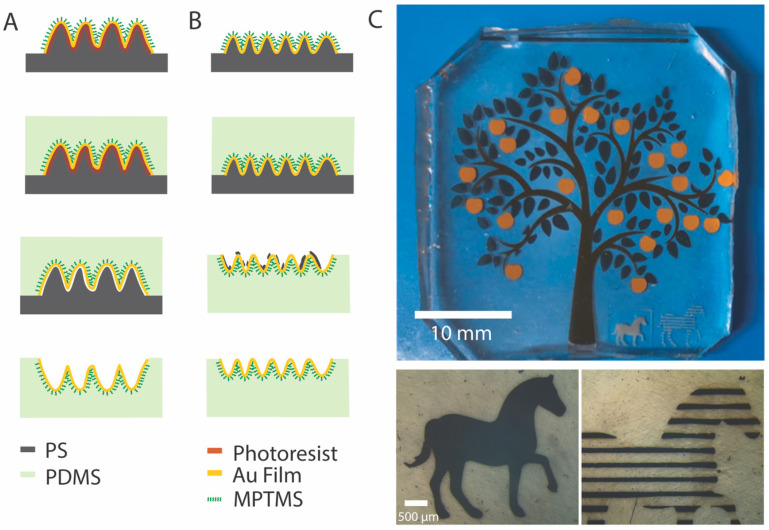
Schematic of two solvent-assisted processes to transfer wrinkled films from PS to PDMS. (**A**) Shrunken electrode with photoresist underneath as sacrificial layer. The surface of the wrinkled thin film was treated with MPTMS, and PDMS was cast onto the treated films and cured. The sacrificial layer was dissolved in acetone, completing the transfer of the wrinkled thin film to PDMS. (**B**) Shrunken electrode with no sacrificial layer on PS substrate. The surface of the wrinkled thin film was treated with MPTMS and PDMS was cast onto the treated films and cured. Incubation in acetone was used to swell and partially remove the PS, leaving some residues on the wrinkled surface. Washing with toluene was then used to remove the residues. (**C**) Thin films transferred onto PDMS with different compositions and thicknesses form an apple tree and horse scene: trunk—5 nm ITO/5 nm Au/5 nm ITO, leaves—1.3 nm ITO/1.3 nm Au/1.3 nm ITO, apples—50 nm Au, horses—20 nm SiO_2_.

Two solvent-assisted methods to transfer wrinkled thin films onto elastomeric substrates, one with a sacrificial layer and another without, were compared to evaluate the advantages and disadvantages of each approach. Figure 1 shows a schematic representation of the two processes tested to transfer wrinkled thin films (A and B) as well as a photo of sample patterned films transferred onto PDMS (C). Figure 1A shows the transfer process with a sacrificial layer of photoresist. To transfer the film with a sacrificial layer, a 400 nm-thick photoresist layer was spin-coated onto vinyl-masked PS prior to gold film deposition. The addition of the photoresist layer increased the wrinkle size compared to wrinkles formed on 20 nm-thick gold films deposited directly onto the PS substrate. This is because the wavelength of the wrinkles is proportional to the combined thickness of the stiff film and the sacrificial layer, and because the sacrificial layer can soften and flow during the shrinking process [11,16,17].

A strong bond between the thin films and the elastomer substrates is essential to impart the robustness and reliability needed for stretchable electronics. However, the bare gold film interacts very weakly with PDMS. To ensure strong adhesion between the wrinkled thin film and the elastomer, MPTMS was used to functionalize the gold surface. MPTMS is a molecular adhesion promoter that can form self-assembled monolayers and has two types of terminal groups with different functionalities. The three methoxy (-OCH_3_) groups can covalently bind to the surface of PDMS and the thiol (-SH) can bond to the gold surface. Thus, when a PDMS base and crosslinker mixture was cast onto the functionalized surface of the shrunken electrode and cured at 60 °C for 4 h, the two materials became strongly bonded. Once the elastomer was cured, it was cut along the edge of the PS to expose the PS/PDMS interface. The PS with cured PDMS was then immersed in an acetone bath to dissolve the photoresist between the wrinkled Au film and the PS. The wrinkled films could be fully lifted off from the PS after only 1 h in acetone, while remaining adhered to the PDMS. 

Figure 1B illustrates the transfer process that involves no sacrificial layer. In this example, a 20 nm-thick Au film was directly sputtered onto a PS substrate. In contrast to Figure 1A, the wavelength of the bare Au film (<1 µm) is much smaller than that of the 20 nm-thick Au film with a layer of photoresist (~4 µm). The wrinkled Au surface was also functionalized with MPTMS and PDMS was cast and cut using the same procedure as above. The cured sample of PS/Au/PDMS was put in an acetone bath for ~6 h. During this time, the PS substrate softened and swelled, and could be removed from the PDMS substrate. However, because of the softening of the PS, residue was left on the surface of the Au film. Since PS is more readily solubilized in toluene than acetone, as a last step, toluene was used to remove residue on the surface of the wrinkled film. Many toluene rinsing steps (~40) were needed to ensure that the rough wrinkled surface was devoid of any PS residue. Although toluene is useful to remove residual PS from the Au films, it causes swelling of PDMS by ~30% [18]. Swelled PDMS is, however, capable of shrinking back to its original dimensions by removing the solvent in a vacuum or through evaporation in an oven at 60 °C. The swelling introduced about 5% strain onto the wrinkled film and no cracks or delamination were observed on the transferred films. 

To showcase the potential of transferring electronic devices with complexity by one of the methods described above, a pattern comprised of various materials in different shapes and layout was transferred. Figure 1C shows structured films transferred to PDMS made from various material layers and with different thicknesses: trunk of the apple tree—5 nm ITO/5 nm Au/5 nm ITO, leaves—1.3 nm ITO/1.3 nm Au/1.3 nm ITO, apples—50 nm Au, horses—20 nm SiO_2_. In addition to different film thicknesses and compositions, the pattern transferred is complex and shows dimensions across different scales, from the base of the tree representing a couple millimeters and the stripes of the horse being ~120 µm in width. These films were transferred simultaneously and with no sacrificial layer, demonstrating the ability of this method to transfer different materials, hybrid structures, and multiscale complex patterns. This method is thus straightforward and high-fidelity, enabling the fabrication of stretchable thin film devices comprised of different materials and hybrid structures. 

**Figure 2:**
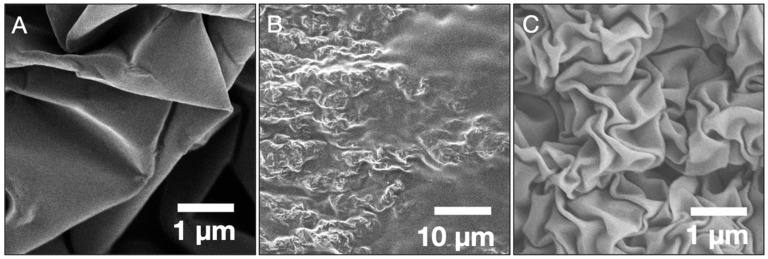
Scanning electron microscope images show the differences arising from of the two transferring methods. (**A**) Topography of a 20 nm-thick Au wrinkled film transferred onto PDMS using a sacrificial photoresist layer. Topography of a 20 nm-thick wrinkled gold film transferred without photoresist onto PDMS (**B**) with polystyrene residue after the acetone bath step and (**C**) after rinsing with toluene.

Both transfer methods have advantages and disadvantages based on the simplicity of the process and fidelity of the transferred patterns. The method using a sacrificial photoresist layer is simpler and more efficient, involving only the direct dissolution of photoresist with acetone, with no PS residue observed via SEM. However, the wavelength of the wrinkles is much larger compared to that of samples with similar Au film thickness transferred with the method devoid of photoresist. Figure 2A shows the surface topography of a 20 nm-thick gold wrinkled film transferred onto PDMS with a sacrificial layer. The increase in wrinkle size arises primarily from the greater combined film thickness (~400 nm for Au + photoresist versus 20 nm for Au only) as previously mentioned. The transfer process without sacrificial layer is longer and requires an extra toluene rinsing step to remove PS residue after the acetone bath. The residue, which can be seen in Figure 2B, fills the valleys on the wrinkled surface after removing the softened PS, but can be effectively eliminated with toluene (Figure 2C shows the same sample as Figure 2B after toluene rinsing). Therefore, the transfer method can be selected depending on the material requirements and the desired application. With the sacrificial layer, the transfer is fast and requires less post-transfer cleanup, but produces larger wrinkles whereas without the sacrificial layer, the transfer requires more time and an extra toluene rinsing stage, however the result is smaller wrinkled features. Each transfer method could prove useful depending on the requirements of roughness (wrinkle size), tolerance to PS residue and swelling, and compatibility with toluene for the stretchable electronic device.

### 3.2. Multi-Material Thin Film Transfer

Most stretchable thin film devices or integrated circuits require the incorporation of multiple materials into layers with dimensions going from the macro to the microscale and functions ranging from conductor to dielectric. Therefore, it is essential that transfer methods can accommodate different materials and structures simultaneously. This would significantly simplify stretchable electronic device fabrication, achieve better adhesion between the different layers, and solve interconnection problems between different parts of the device, as well as prove useful for the fabrication of large area wearable electronic arrays. 

**Figure 3:**
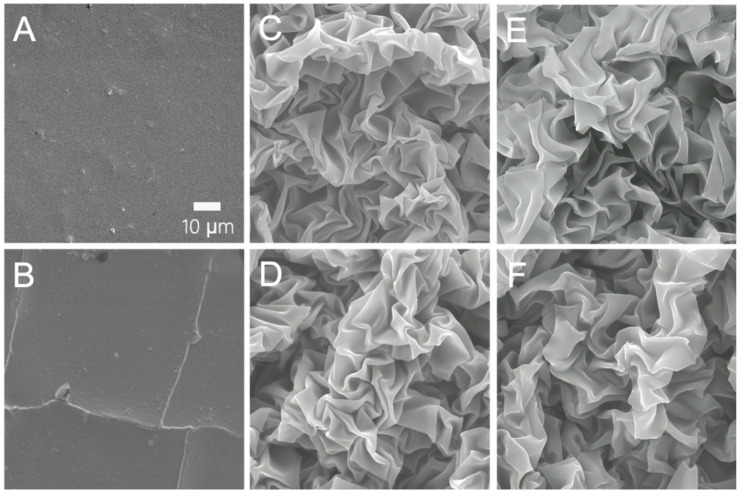
Surface topography of 5 nm ITO/5 nm Au/5 nm ITO multilayer films and 20 nm SiO_2_ films on PS and PDMS substrates. (**A**) Flat ITO/Au/ITO film on PS. (**B**) Flat ITO/Au/ITO film transferred onto PDMS. (**C**) Wrinkled ITO/Au/ITO film on PS. (**D**) Wrinkled ITO/Au/ITO film on PDMS. (**E**) Wrinkled 20 nm SiO_2_ on PS. (**F**) Wrinkled 20 nm SiO_2_ on PDMS.

To this end, we tested the ability of the direct transfer method to transfer composite layered thin films. SEM images of flat and wrinkled 5 nm ITO/5 nm Au/5 nm ITO film on PS substrates are shown in Figure 3A,C, respectively. The same films transferred onto PDMS are shown in Figure 3B,D, respectively. When measuring the resistance of transferred films, wrinkled films on PDMS showed the same values as on the rigid PS substrate. On the other hand, the transferred flat films (Figure 3B) were not electrically conductive due to extensive cracking. Both the morphology and electrical measurements suggest that the wrinkled structures make the thin film more tolerant to the strain applied during the transfer process. Figure 3C,D (ITO/Au/ITO films), along with Figure 3E,F (20 nm-thick SiO_2_ on PS and PDMS), highlight the potential of this method for transferring multilayer, high modulus, and low fracture toughness wrinkled films from PS to PDMS. This will bring real benefit to the development of stretchable thin film electronic devices because most devices incorporate specific architectures composed of conductor, dielectric, and insulator materials. The one-time transfer will simplify the fabrication of stretchable devices and circuits, and significantly improve the bonding between different films. Moreover, the wrinkles increase the tolerance of modulus films to external strain during the fabrication, as shown by the comparison between Figure 3B,D. 

### 3.3. Stretchable Thin-Film Heaters

**Figure 4:**
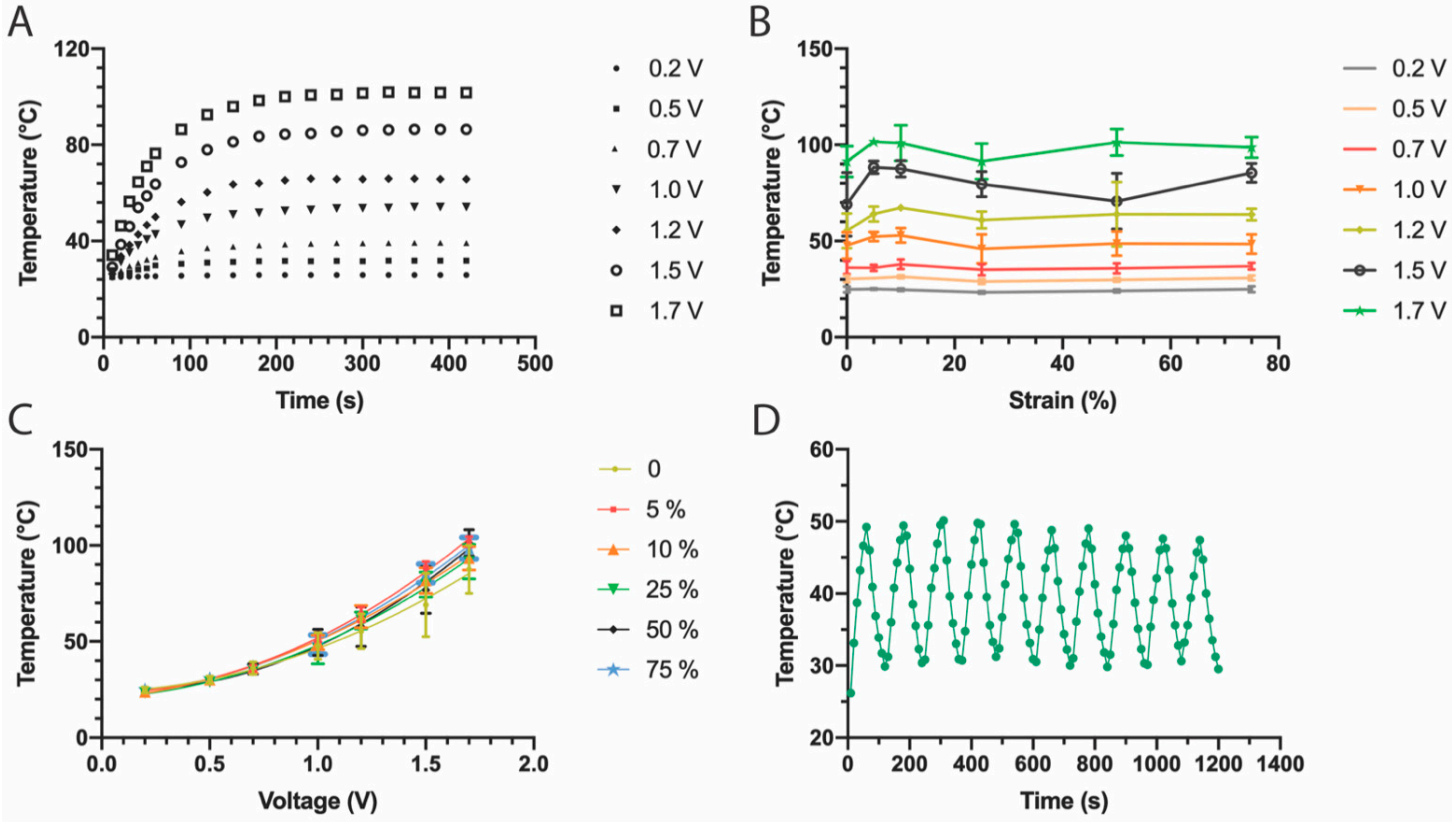
Characterization of stretchable Au heaters. (**A**) Temperature profiles under varying applied voltage (0.2–1.7 V). (**B**) The plateau temperature changes with various voltages but remains similar for any given voltage under different strains. (**C**) Changes in temperature vs. voltage for various applied strains (each series corresponds to an applied strain). (**D**) Cycling on/off response under an applied voltage of 1.5 V shows the reproducibility of the heating and cooling cycles.

As proof-of-concept of the fabrication of stretchable devices through solvent-assisted transfer of structured thin films, stretchable thin film heaters were fabricated. Two types of heaters were built: heaters with a single layer of Au and heaters with multiple layers, composed of ITO/Au/ITO. The heaters were made containing a resistive heating pad (3 × 1.5 cm) and two small contact pads (0.5 × 0.5 cm). To characterize the mechanical and thermal properties of the stretchable heaters, thermal paste was applied on the surface of the resistive heating element, and the temperature of the thermal paste was monitored for various applied input voltages (Figure S1). The temperature vs. time profile for Au heaters under various applied voltages (0.2–1.7 V) over 420 s is shown in Figure 4A. At first the temperature of the thermal paste increases rapidly, especially during the first 60 s, then slows down and reaches a plateau (or stable temperature) within 100–200 s. Within the first 60 s, the temperature increases almost linearly, with heating rates for Au heaters of 0.25 °C/s and 0.70 °C/s under a bias of 1.0 V and 1.7 V, respectively. When the input voltage is below 1 V, the plateau temperature is reached faster than with higher applied voltages, as expected from the smaller change in temperature from room temperature. From the temperature-time profile it can be seen that the temperature of the heater could reach and maintain a stable temperature within minutes. 

To investigate the stretchability of the structured Au film resistive elements, the heater was operated under various applied tensile strains at constant voltage and the plateau temperatures were measured. This was repeated for various applied voltages and the results are summarized in Figure 4B,C. The plot of temperature vs. strain (Figure 4B) shows mostly uniform horizontal lines for every applied voltage, indicating that Joule heating is stable when the heater is stretched. Figure 4C shows the same data, but now presented as a temperature vs. voltage plot. In this plot, it can be clearly observed that the heater presents a non-linear, quadratic (Figure S2), response to voltage. This functional dependence is maintained even at different applied strains, although higher voltages (higher temperatures) produce higher variability. This is expected as the heat can be quickly dissipated and small changes in the conductivity of the heating element can have a strong influence on the recorded temperature. It is worth noting that the temperatures recorded for strains at 5% show a slightly higher temperature compared to the temperature recorded under no applied strain. This can be explained by the way the stretchable heater is fixed in the stretching setup; when the thin film on the PDMS substrate was fixed on the stretcher and the screws that clamped the substrates were tightened, the PDMS substrate was squeezed, causing the film to undergo a small amount of compressive bending. The film was subsequently flattened during stretching, which means that the 0% strain state resulted in a slightly bent film and 5% strain state more accurately represented a relaxed “0% strain” state. To further check the temperature reproducibility, on-off cycles were applied to the heaters every 60 s under an applied voltage of 1.5 V (Figure 4D). The thin film heaters showed highly reproducible behavior, reliable performance, and fast heating response as indicated by the uniformity of the on/off response curve. 

**Figure 5:**
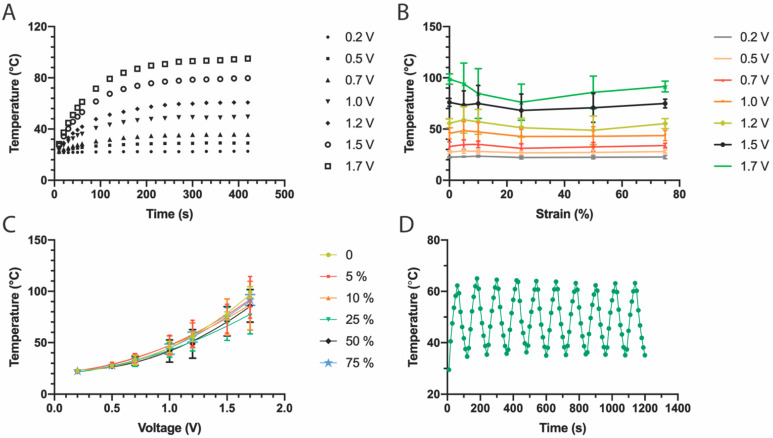
Characterization of stretchable ITO/Au/ITO heaters. (**A**) Temperature profiles under varying applied voltage (0.2–1.7 V). (**B**) The plateau temperature changes with various voltages but remains similar for any given voltage under different strains. (**C**) Changes in temperature vs. voltage for various applied strains (each series represents a given strain value). (**D**) Cycling on/off response under an applied voltage of 1.5 V shows the reproducibility of the temperature reached at the plateau.

To investigate the applicability of our solvent-assisted transfer fabrication of resistive heaters to composite ceramic/metal multilayered films, ITO/Au/ITO thin film heaters were fabricated and characterized. Although the heating element is comprised of ITO/Au/ITO multilayers, the contact pads were directly connected to the central Au heating layer. This fabrication step is very important to prevent the heater from burning because of a high contact resistance that could lead to overheating of the contact area. The composite thin film heaters were characterized in the same way as the Au heaters described above. Overall, similar thermal and mechanical characteristics were observed as with the Au heaters (Figure 5): fast and reproducible heating kinetics, excellent stretchability, and no noticeable deterioration over on/off cycling. However, some minor differences were observed between the two types of heaters. The temperature-time profile of composite heaters (Figure 5A) shows a slightly lower stable temperature and slower heating rate for each applied voltage. The temperature increases at a rate of 0.20 °C/s and 0.55 °C/s under 1.0 V and 1.7 V, respectively. This difference is attributed to the difference in heat transfer between the central Au resistive layer and the 5 nm ITO layer, which ultimately limits heat transfer to the thermal paste. The temperature versus strain profiles (Figure 5B,C) indicate that the multilayer thin film heaters have slightly less reproducibility as evidenced by the larger error bars obtained from replicate devices, especially at higher applied voltages. The lower reproducibility of the multilayer heaters at higher voltages could be caused by the two 5 nm ITO layers that are more brittle and could lead to some cracking during the stretching process. 

It is worth mentioning that the ITO/Au/ITO multilayer heater is representative of an electronic device with a hybrid structure incorporating various materials (metal and ceramic); together with the contact pads which interconnect with other components, they comprise a unit cell that can be integrated into an array or a more complex circuit. This fabrication approach can be further extended to encompass other device components like resistors, capacitors, inductors, transistors, and even a hybrid network of them can be realized. The simultaneous transfer of multiple device components would also address interconnection issues that arise for stretchable electronic arrays, leading to improved long-term stability. 

## 4. Conclusions

This work demonstrates a method for the fabrication of thin film stretchable devices by solvent-assisted transfer of wrinkled thin films from rigid substrates to elastomeric PDMS. Wrinkled thin films were obtained by shrinking flat films patterned on and supported by a shape memory polymer. The transfer process involves modifying the surface of the wrinkled film with an adhesion promoter that forms a chemical bond between PDMS and the film, and the subsequent release of the structured film through dissolution of a sacrificial layer or softening of the PS substrate. Structured films transferred using these two approaches were characterized and compared. The solvent-assisted transfer process ensures the faithful transfer of the wrinkled structures with millimeter to micrometer dimensions in the stretchable devices.

A proof-of-concept application was demonstrated by fabricating and characterizing stretchable thin film Au and ITO/Au/ITO multilayer stretchable heaters. The fabrication of highly stretchable heaters is simple, and since the connection pads and the heating elements were already overlayed, the transfer approach results in very strong adhesion between all elements of the heater. To measure thermal properties and stretchability of the heaters, the temperature of thermal paste in contact with the heaters was monitored at various applied strains and voltages. The results show that both types of stretchable thin films have rapid Joule heating kinetics, and the temperature can reach more than 50 °C with only 1 V of applied voltage. The heaters also displayed high stretchability with nearly constant performance even at 75% applied strain, and high reproducibility when subjected to on/off duty cycles. Overall, the transfer process is a simple and effective method for fabricating hybrid materials and structures for stretchable electronic devices. 

## Data Availability

The data presented in this study are available on request from the corresponding author.

## Supplementary Materials

The following supporting information can be downloaded at: https://www.mdpi.com/article/10.3390/mi13020334/s1, Figure S1: Schematic of the characterization of stretchable Au heaters and ITO/Au/ITO heaters; Figure S2: Non-linear regression performed showing the quadratic fits presented in Figure 4C and Figure 5C for stretchable Au heaters and ITO/Au/ITO heaters.
