# Supplementary material for: Efficient Multi-Material Structured Thin Film Transfer to Elastomers for Stretchable Electronic Devices
Source: Micromachines (Basel). 2022 Feb 20;13(2):334. doi: 10.3390/mi13020334 (PMC8879079; doi:10.3390/mi13020334)
Supplement: Supplementary file 1 [file micromachines-13-00334-s001.zip › micromachines-1592046-SI.pdf]

micromachines

MDPI

Article

Supplemental Information: Efficient Multi-Material Structured Thin Film Transfer to Elastomers for Stretchable Devices

Xiuping Ding [1], Jose M. Moran-Mirabal [1,2,*]

[1] Department of Chemistry & Chemical Biology, McMaster University, 1280 Main Street West, Hamilton, ON L8S 4M8, Canada
[2] Brockhouse Institute for Materials Research, McMaster University, 1280 Main Street West, Hamilton, ON L8S 4M8, Canada
* Correspondence: mirabj@mcmaster.ca

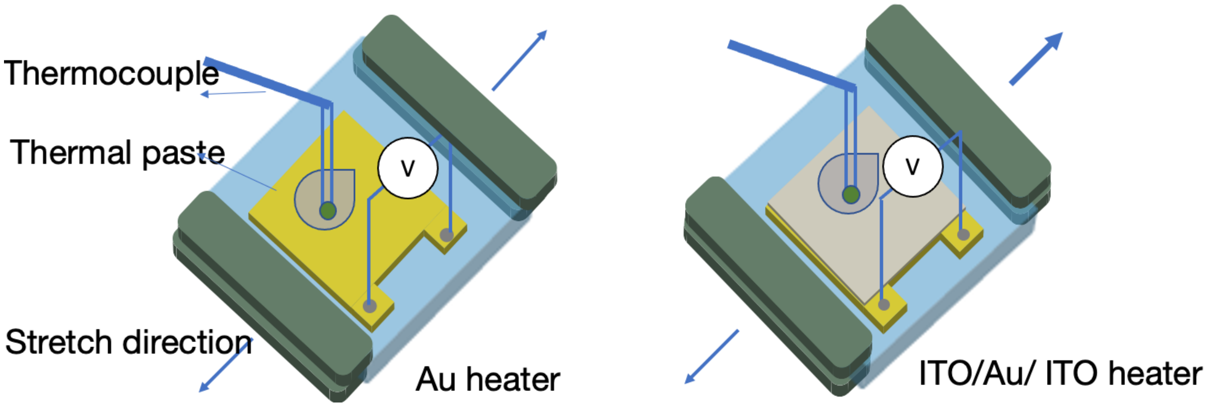

Figure S1. Schematic of the characterization of stretchable Au heaters and ITO/Au/ITO heaters

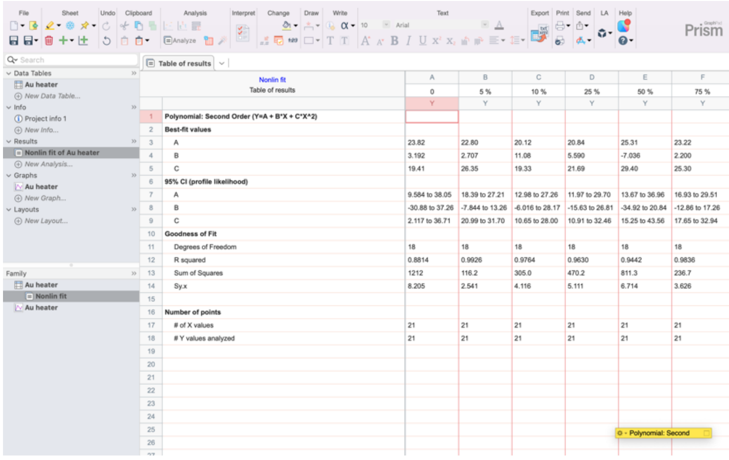

Nonlin fit Table of results	A 0	B 5 %	C 10 %	D 25 %	E 50 %	F 75 %
Polynomial: Second Order (Y=A + B*X + C*X^2)						
Best-fit values						
A	23.82	22.80	20.12	20.84	25.31	23.22
B	3.192	2.707	11.08	5.590	-7.036	2.200
C	19.41	26.35	19.33	21.69	29.40	25.30
95% CI (profile likelihood)						
A	9.584 to 38.05	18.39 to 27.21	12.98 to 27.26	11.97 to 29.70	13.67 to 36.96	16.93 to 29.51
B	-30.88 to 37.26	-7.844 to 13.26	-6.016 to 28.17	-15.63 to 26.81	-34.92 to 20.84	-12.86 to 17.26
C	2.117 to 36.71	20.99 to 31.70	10.65 to 28.00	10.91 to 32.46	15.25 to 43.56	17.65 to 32.94
Goodness of Fit						
Degrees of Freedom	18	18	18	18	18	18
R squared	0.8814	0.9926	0.9764	0.9630	0.9442	0.9836
Sum of Squares	1212	116.2	305.0	470.2	811.3	236.7
Sy.x	8.205	2.541	4.116	5.111	6.714	3.626
Number of points						
# of X values	21	21	21	21	21	21
# Y values analyzed	21	21	21	21	21	21

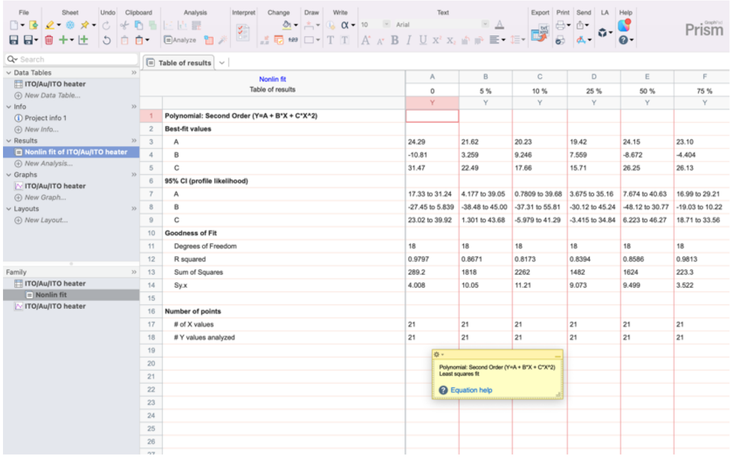

Nonlin fit Table of results	A 0	B 5 %	C 10 %	D 25 %	E 50 %	F 75 %
Polynomial: Second Order (Y=A + B*X + C*X^2)						
Best-fit values						
A	24.29	21.62	20.23	19.42	24.15	23.10
B	-10.81	3.259	9.246	7.559	-8.672	-4.404
C	31.47	22.49	17.66	15.71	26.25	26.13
95% CI (profile likelihood)						
A	17.33 to 31.24	4.177 to 39.05	0.7809 to 39.68	3.675 to 35.16	7.674 to 40.63	16.99 to 29.21
B	-27.45 to 5.839	-38.48 to 45.00	-37.31 to 55.81	-30.12 to 45.24	-48.12 to 30.77	-19.03 to 10.22
C	23.02 to 39.92	1.301 to 43.68	-5.979 to 41.29	-3.415 to 34.84	6.223 to 46.27	18.71 to 33.56
Goodness of Fit						
Degrees of Freedom	18	18	18	18	18	18
R squared	0.9797	0.8671	0.8173	0.8394	0.8586	0.9813
Sum of Squares	289.2	1818	2262	1482	1624	223.3
Sy.x	4.008	10.05	11.21	9.073	9.499	3.522
Number of points						
# of X values	21	21	21	21	21	21
# Y values analyzed	21	21	21	21	21	21

Figure S2. Non-linear regression performed showing the quadratic fits presented in Figures 4C and 5C for stretchable Au heaters and ITO/Au/ITO heaters
